# MicroRNA Signature in Renal Cell Carcinoma

**DOI:** 10.3389/fonc.2020.596359

**Published:** 2020-11-30

**Authors:** Soudeh Ghafouri-Fard, Zeinab Shirvani-Farsani, Wojciech Branicki, Mohammad Taheri

**Affiliations:** ^1^ Urology and Nephrology Research Center, Shahid Beheshti University of Medical Sciences, Tehran, Iran; ^2^ Department of Cell and Molecular Biology, Faculty of Life Sciences and Technology, Shahid Beheshti University G.C., Tehran, Iran; ^3^ Malopolska Centre of Biotechnology of the Jagiellonian University, Kraków, Poland; ^4^ Urogenital Stem Cell Research Center, Shahid Beheshti University of Medical Sciences, Tehran, Iran

**Keywords:** miRNA, renal cell carcinoma, expression, cancer, biomarker

## Abstract

Renal cell carcinoma (RCC) includes 2.2% of all diagnosed cancers and 1.8% of cancer-related mortalities. The available biomarkers or screening methods for RCC suffer from lack of sensitivity or high cost, necessitating identification of novel biomarkers that facilitate early diagnosis of this cancer especially in the susceptible individuals. MicroRNAs (miRNAs) have several advantageous properties that potentiate them as biomarkers for cancer detection. Expression profile of miRNAs has been assessed in biological samples from RCC patients. Circulatory or urinary levels of certain miRNAs have been proposed as markers for RCC diagnosis or follow-up. Moreover, expression profile of some miRNAs has been correlated with response to chemotherapy, immunotherapy or targeted therapeutic options such as sunitinib. In the current study, we summarize the results of studies that assessed the application of miRNAs as biomarkers, therapeutic targets or modulators of response to treatment modalities in RCC patients.

## Introduction

Renal cell carcinoma (RCC) is the 15^th^ most frequent cancer, based on the statistics provided by GLOBOCAN (1). This kind of cancer includes 2.2% of all diagnosed cancers and 1.8% of cancer-related mortalities (1). The incidence of this type of cancer is different in different regions. RCC is associated with numerous risk factors among them are smoking, obesity, and hypertension (2). The varied incident and mortality rates of RCC in different geographical regions necessitate enactment of regional screening programs and development of precise biomarkers (2). Among the screening methods for sporadic RCC, urine dipstick has yielded low level of accuracy impeding its clinical application (3). Moreover, none of the proposed serum and urine markers such as aquaporin 1, perilipin 2, and KIM1 had enough sensitivity or specificity to be applied in this regard (4). On the other hand, computed tomography and abdominal ultrasound suffer from high cost and low sensitivity for the identification of small tumors, respectively (2, 3). Therefore, development of effective non-invasive screening methods for RCC is a necessity. Recent investigations have potentiated microRNAs (miRNAs) as screening tools for several kinds of human malignancies (5). These transcripts contribute in the pathogenesis of human disorders. In this review, we clarify the main points of studies in RCC to judge the potential of miRNAs as biomarkers or therapeutic targets in this malignant condition.

### miRNA Biogenesis and Function

miRNAs have sizes about 23 nucleotides and are present in different species. By acting as antisense transcripts, miRNAs post-transcriptionally decrease expression of their targets. Although the regulatory effects of each miRNA on the expression of its target gene is not great, the resultant interactive network between miRNAs, target genes and downstream effectors plays crucial impacts on the regulation of cellular functions (6). The majorities of these transcripts are transcribed from DNA templates into primary miRNAs and undergo a number of steps to be processed into the precursor and mature miRNAs, respectively (7). Two kinds of RNase III molecules, i.e., Drosha and Dicer proteins participate in the miRNA processing in the nuclear and cytoplasmic cellular compartments, respectively (7). The critical function of miRNAs in gene expression modulation is additionally highlighted by the point that an individual gene is concurrently regulated by several miRNAs, and each miRNA can modulate expression of several targets which have sequence complementarity with its seed region (8). About one-third of human genome and virtually all essential cell processes are expected to be regulated by miRNAs (9, 10). The role of miRNAs in the pathogenesis of human cancers has been vastly examined (11). These molecules have been reported to influence the main features of carcinogenic process such as sustained proliferative capacity, evasion from growth inhibitor signals, resistance to apoptosis, induction of invasive and metastatic programs, and enhancement of angiogenic processes (12). The importance of miRNAs in development of cancer has been firstly highlighted through the spotting miR-15a and miR-16-1 in a commonly deleted region in B-cell chronic lymphocytic leukemias (13). Subsequent investigations revealed other genomic alterations in a number miRNA coding genes in different cancers such as lung cancer (14), melanoma as well as ovarian and breast cancers (15). Moreover, well-known oncogenes such as c-Myc were shown to influence expression of oncogenic activates miRNAs including miR-17-92 (16) or inhibit expression of tumor suppressor miRNAs including miR-15a, miR-26, miR-29, miR-30, and let-7 (17). In RCC, quite a lot of investigation have measured expression profile of miRNAs in different biological samples to identify the pathogenic roles of these transcripts in the development of this type of cancer (18).

### Dysregulated miRNAs in RCC

**Figure 1:**
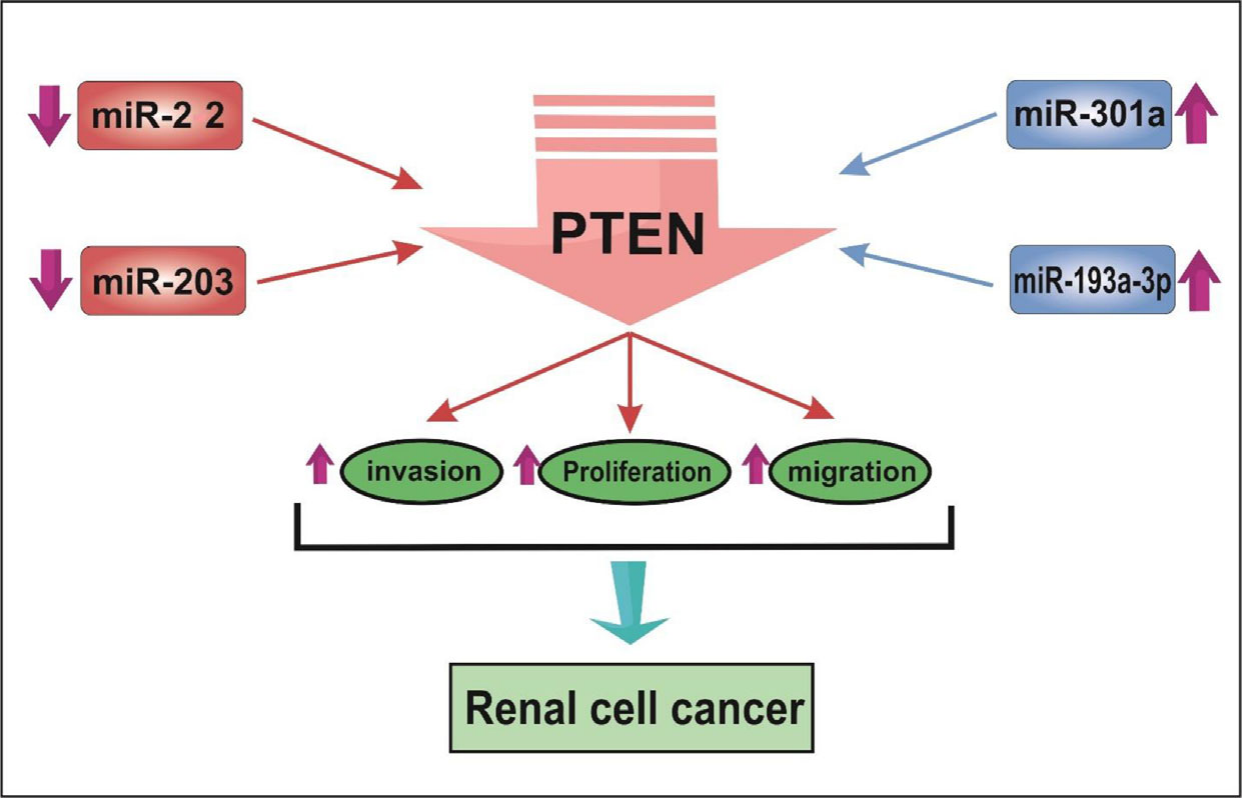
The schematic depiction of the interplay between miRNAs and tumor-suppressive gene PTEN in renal cell cancer. MiR-22 and miR-203 are decreased, while miR-301 and miR-193a-3p are up-regulated in RCC. miRNA expression changes result in reducing the expression of PTEN. Consequently, cell proliferation, invasive behavior, and migration are enhanced in RCC.

A number of studies have assessed differentially expressed miRNAs and their target genes in RCC samples and normal control. Using this approach, Li et al. have identified down-regulation of 521 genes and up-regulation of 473 genes in RCC samples. Protein-protein interaction network showed RHCG, RALYL, SLC4A1, UMOD, and CA9 as nodes with high degrees of interactions. The differentially expressed genes were enriched in cytokine and cytokine receptor pathway (19). Such approaches are useful in identification of biomarkers and therapeutic targets for RCC. Other studies have reported dysregulation of a number of miRNAs in RCC samples. **Figure 1** shows a number of dysregulated miRNAs in RCC and their interaction with the PTEN tumor suppressor.

The following sections describe the function of these miRNAs.

### Up-Regulated miRNAs in RCC

Numerous oncomiRs have been recognized in RCC. Gottardo et al. have described up-regulation of miR-28, miR-185, miR-27, and let-7f-2 in tissue samples obtained from RCC patients compared to normal kidney samples. Notably, these miRNAs were different from up-regulated miRNAs in bladder cancer samples in their cohort of patients, implying the presence of distinctive miRNA signature between these two cancers of the urogenital system (20). Wulfken et al. have investigated miRNA signature in both tissue and serum specimens of patients with RCC. They reported over-expression of 109 circulatory miRNAs in cancer patients; among them were 36 miRNAs that were up-regulated in tissue samples as well. Additional verification steps indicated up-regulation of miR-1233 in another cohort of RCC patients. Notably, expression patterns of this miRNA in patients with angiomyolipoma or oncocytoma was similar with RCC patients (21). miR-301a is another up-regulated miRNA in RCC cell lines and clinical samples. Over-expression of this miRNA has been associated with advanced stage and poor survival of RCC patients. Mechanistically, miR-301a has been displayed to target PTEN tumor suppressor (22). Two other oncomiRs, namely, miR-22 and miR-193a-3p also suppress expression of PTEN in RCC cells (23, 24). In addition, miR-1293 has been up-regulated in RCC cells enhancing viability of these cells their migratory potential and invasiveness. These effects are mediated through suppression of Hydrocyanic Oxidase 2 (25). **Table 1** gives a summary of the roles of up-regulated miRNAs in RCC.

**Table 1:** Up-regulated miRNAs in RCC (ANTTs: adjacent non-tumoral tissues).

miRNA	Samples	Targets/Regulators	Pathways	Roles	Ref
miR-301a	516 tumor samples and 71 ANTTs	PTEN	cell cycle G1/S transition	miR-301a regulates PTEN expression.	(22)
miR-429	28 pairs of tumor and ANTTs	CRKL	TGF-β، SOS1/MEK/ERK/MMP2/MMP9 pathway	Has a role in migration and invasion	(26)
miR-92a-3p	16 pairs of RCC tissues and ANTTs	FBXW7	-	miR-92a-3p silencing suppressed cell proliferation and reduced colony number.	(27)
miR-1293	PRCC (292 tumor tissues and 34 normal) and ccRCC (545 tumor tissues and 71 normal), from TCGA database	HAO2	EMT	Has a role in cell viability, invasion, and migration	(25)
miR-210-3p	21 paired ccRCC tissues and ANTTs and urine samples	-	f VHL/hypoxia 314 – VEGFR	-	(28)
miR-671-5p	90 primary ccRCC tissues and 90 ANTTs	APC	Wnt signaling	Has a role in invasion and migration	(29)
miR-935	Twenty-five patient samples with ccRCC/Cancer tissues and normal kidney tissues were frozen	IREB2	-	Has a role in proliferation, migration and invasion	(30)
miR-592	114 paired ccRCC tissues and ANTTs and urine samples	SPRY2	-	Has a role in proliferation, migration and invasion	(31)
miR-22	480 paired ccRCC tissues and ANTTs and urine samples	PTEN	-	Has a role in invasion	(23)
miR-720	30 paired cancer tissues and ANTTs	E-cadherin and E-catenin		Has a role in EMT and metastasis	(32)
miR-210, miR-218, and miR-1233	Plasma samples from 54 RCC patients and 50 healthy individuals	-	-	Patients with up-regulated miR-210, miR-221 and miR-1233 had higher risk of specific death by RCC.	(33)
miR-122	148 cancer tissues and 60 ANTTs	Dicer	miR‐122/Dicer pathway	miR‐122 induces EMT, migration and invasion in RCC.	(34)
miR‐125b	276 paired cancer tissues and ANTTs	-	-	miR‐125b forecasts recurrence and outcome of ccRCC after surgical resection.	(35)
miR-378 and miR-210	Serum samples from 195 RCC patient and 100 healthy controls	-	-	Combination of MiR-378 and MiR-210 Serum Levels serve as powerful non-invasive Detection in RCC.	(36)
miR-224	20 paired cancer tissues and ANTTs and serum sample from 108 ccRCC patients	-	-	miR-224 increased cell viability and invasion ability, reduced apoptosis.	(37)
miR‐7	72 paired samples from cancer tissues and ANTTs	MEG3, RASL11B	-	miR‐7 induces progression of ccRCC.	(38)
miR-203a	40 paired cancer tissues and ANTTs	GSK-3β	Wnt/β-catenin pathway	miR-203a induces cell proliferation, migration, cell cycle, and suppresses apoptosis of RCC cells.	(39)
miR-155	20 paired cancer tissues and ANTTs	FOXO3a	-	miR-155 increased the proliferation, and inhibited apoptosis and cell cycle arrest.	(40)
miR-125b	24 paired cancer tissues and ANTTs	-	-	miR-125b induced cell mobility and inhibited apoptosis.	(41)
miR-122	90 paired cancer tissues and ANTTs	Occludin	MAPK pathway	miR-122 enhanced cell proliferation, migration and invasion.	(42)
miR-221/222	57 paired cancer tissues and ANTTs	KDR	angiogenesis pathways	miR-221/222 enhances tumor cell proliferation.	(43)
miR-223‐3p	156 nephrectomy and 46 kidney biopsy specimens	-	-	Levels of miR-223‐3p may be biomarker for ccRCC and it was correlated with cancer‐specific survival.	(44)
miR-21, miR-155, and/or miR-142-5p	59 normal kidney and 54 tumor specimens; and 38 paired cancer tissues and ANTTs	-	-	Three-miRNA combination is as a potential predictor of renal cancer in patients.	(45)
miR-193a-3p	30 paired cancer tissues and ANTTs	PTEN	PI3K signaling pathway	miR-193a-3p induces cell proliferation, cell migration and the cell cycle.	(24)
miR‐193a‐3p and miR‐224	30 paired cancer tissues and ANTTs	ST3GalIV	PI3K/Akt pathway	MiR‐193a‐3p and miR‐224 enhanced RCC cell proliferation and migration by directly suppressing ST3GalIV.	(46)
miR-99, miR-miR-200b,miR-106a, miR-106b	56 paired cancer tissues and ANTTs	mTOR, VHL		These miRNAs increased the aggressiveness of RCC.	(47)
miR-106b-5p	20 paired cancer tissues and ANTTs	β-catenin, LZTFL1, SFRP1 and DKK2	Wnt/β-catenin signaling	miR-106b-5p induces tumor growth and metastasis through induction of Wnt/β-catenin signaling.	(48)
hsa-miR-27b, hsa-miR-23b and hsa-miR-628-5p	123 paired cancer tissues and ANTTs	c-Met and Notch1	-	These miRNAs may be biomarkers of sunitinib response.	(49)
miR-9-1	48 paired cancer tissues and ANTTs	-	-	MiR-9-1 induces ccRCC progression.	(50)
miR-193a-3p, miR-362 and miR-572	Serum from 107 RCC patients and 107 controls	-	-	These miRNAs may be diagnostic biomarker for RCC.	(51)
miR-34a	85 paired cancer tissues and ANTTs	MET, E2F3, TP53INP2 and SOX2	-	miR-34a promotes RCC tumorigenesis and progression.	(52)
miR-210	284 (264 primary and 20 metastatic ccRCC) paired cancer tissues and ANTTs	-	-	miR-210 induces aggressive behavior in ccRCC.	(53)
miR-122	40 paired cancer tissues and ANTTs	Sprouty2		miR-122 induces cell proliferation by targeting Sprouty2.	(54)
miR-146a-5p, miR-128a-3p, and miR-17-5p	30 tissue samples of ccRCC (10 non-malignant 20 tissue samples of primary ccRCC),	CXCL8/IL8, UHRF1, MCM10, and CDKN3	-	These miRNAs induce the evolution from primary RCC without metastases into metastatic form.	(55)
miR-106b-5p	40 paired cancer tissues and ANTTs	SETD2	P53 pathway	miR-106b-5p induces cells proliferation and inhibits apoptosis through reducing of SETD2 expression.	(56)
miR-144-3p	Tissues from 60 patients with ccRCC, 8 patients with nccRCC and 10 patients with renal hamartoma	ARID1A	-	mir-144-3p induces cell Proliferation and metastasis, in ccRCC by reducing ARID1A expression.	(57)
miR-1233	paired sample tissue and serum samples from 30 patients	-	-	These miRNAs may be useful as diagnostic biomarkers.	(58)
miR-29b	45 paired cancer tissues and ANTTs	KIF1B	-	miR-29b increases cell proliferation and invasion, and suppresses apoptosis.	(59)
miR-210-3p	15 paired cancer tissues and ANTTs	TWIST1	EMT pathway	miR-210-3p promotes cell proliferation and tumorigenesis.	(60)
miR-210 and miR-1233	Serum samples from 82 ccRCC patients and 80 healthy controls	-	-	miR-210 and miR-1233 might be useful as liquid biopsies for diagnosing RCC patients.	(61)
miR-18a-5p	42 paired cancer tissues and ANTTs	-	-	miR-18a-5p enhances cell proliferation and cell mobility, and reduces cell apoptosis.	(62)
miR-489-3p and miR-630	33 paired cancer tissues and ANTTs	OCT2/c-Myc	-	MiR-489-3p and miR-630 induced chemoresistance to oxaliplatin.	(63)
miR-21	99 paired cancer tissues and ANTTs	-	cell cycle	Has a role in migration, invasion, proliferation, and resistance to apoptosis	(64)
miR-21	104 paired cancer tissues and ANTTs	TIMP3		Decreased miR-21 expression decreased cell invasion and migration and inhibited cells apoptosis.	(65)

### Down-Regulated miRNAs in RCC

In a high throughput approach, Nakada et al. have assessed miRNA signature in clear cell carcinomas (CCCs)., and chromophobe RCC compared with normal kidney tissues. They reported down-regulation of 37 and 51 miRNAs in CCC and chromophobe RCC, respectively. As the number of up-regulated miRNAs in cancer tissues was significantly lower than the number of down-regulated ones, authors have deduced that expression of miRNAs have a tendency to be decreased in both histological types of RCC compared with normal renal samples. miR‐141 and miR‐200c were the most remarkably under-expressed miRNAs in CCC samples being down-regulated in all assessed samples of this type. *In silico* and functional studies indicated that decreased expression of miR‐141 and miR‐200c in CCCs may inhibit CDH1/E‐cadherin expression through increasing ZFHX1B levels (66). Two other tumor suppressor miRNAs, namely, miR-30c-5p and miR-138-1 levels, have been down-regulated in RCC samples even in the early stage tumors. Its expression has been lower in RCC samples of Fuhrman grade G3 + G4 compared with G2 (67). Another commonly down-regulated miRNA in RCC is miR-362-3p. Forced up-regulation of miR-362-3p resulted in the attenuation of cell proliferation, induction of cell cycle arrest and reduction of motility. These effects are exerted through modulation of AKT/FOXO3 signaling. SP1 has been identified as a direct target of miR-362-3p (68). Besides, expression of miR-200b has been reduced in RCC samples. Forced over-expression of miR-200b in the RCC cell lines has inhibited their migration and invasiveness and reduced cancer metastasis in xenograft models. Laminin subunit alpha 4 (LAMA4) has been predicted as a direct target of miR-200b (69). **Table 2** summarizes the data about down-regulated miRNAs in RCC.

**Table 2:** Tumor suppressor miRNAs in RCC.

miRNA	Samples	Targets/Regulators	Signaling Pathways	Roles	Ref
hsa-miR-30c-5p	47 paired tumor samples and ANNTs	-	-	miR-30c-5p inhibits proliferation and tumor formation.	(67)
hsa-miR-138-1		-	-	miR-138-1 might be associated with an unfavorable course of the disease.	(67)
miR-363	77 adjacent normal renal tissues	S1PR1	ERK, including PDGF-A, PDGF-B, EMT	miR-363 inhibited the proliferation, migration and invasive capacity of ccRCC cells.	(70)
miR-362-3p	Twenty-five paired of RCC tissues and ANTTs	SP1	AKT/FOXO3	miR-362-3p inhibited the proliferation of RCC cells.	(68)
miR‐214	-	LIVIN	-	miR‐214 reduces the cell proliferation and tumorigenesis.	(71)
miR-133b	60 paired cancerous tissues and ANTTs	-	ERK	miR-133b suppresses cell proliferation, migration and invasion, while inducing apoptosis.	(72)
miR-206	60 paired cancer tissues and ANTTs	CDK6	-	MiR-206 effectively caused apoptosis and cell cycle arrest at G0/G1 phase.	(73)
miR-143	67 paired ccRCC tissues and ANTTs	ABL2	-	miR-143 decreases cells adhesion, migration and EMT.	(74)
miR-124 and miR-203	34 paired ccRCC tissues and ANTTs	ZEB2	EMT	miR-124 and miR-203 inhibit cell proliferation and migration.	(75)
miR‐101‐5p and miR‐101‐3p	18 clinical ccRCC tissue samples/5 patients resistant to several tyrosine kinase inhibitor	DONSON	G2/M checkpoint, EMT	Expression of miR‐101‐5p induced cell cycle arrest and apoptosis.	(76)
miR-765	36 ccRCC patient samples 18 non-ccRCC patient samples and 18 plasma samples (preoperative and operational day 7),	PLP2	-	Up-regulation of miR-765 inhibited cell proliferation and metastasis.	(77)
miR-212-5p	32 pairs of ccRCC and ANNTs	TBX15	-	miR-212-5p acted as a tumor suppressor gene in ccRCC.	(78)
miR-200 family	23 paired ccRCC tissues and ANTTs and urine samples			miR-200c affects the carcinogenic potential of malignant cells.	(79)
miR-135a-5p	96 paired cancer tissues and ANTTs	-	-	Expression of miRNA-135a-5p can identify renal carcinogenesis and metachronous metastasis in ccRCCs.	(80)
miR-141	20 ccRCC tissues	ZEB2	proliferative pathways	miR-141 expression in ccRCC decreased cell proliferation	(81)
miR-124-3p, -30a-5p and -200c-3p	87 matched ccRCC tissues	CAV1 and FLOT1	-	Up-regulation of all three miRNAs decreased migration and invasion in ccRCC cell lines.	(82)
miR-148a	52 paired cancer tissues and ANTTs	AKT2	Akt pathway	Has a role in cell proliferation, colony formation, migration and invasion	(83)
miR‐766‐3p	75 tumor tissues and 40 normal tissues	SF2	SF2/P‐AKT/P‐ERK signaling pathway	miR‐766–3p suppresses cell‐cycle progression.	(84)
miR-30a-5p	40 paired cancer tissues and ANTTs And 516 ccRCC patients from the TCGA database	ZEB2	-	miR-30a-5p inhibits cell growth, migration and invasion.	(85)
miR-129-3p	69 paired cancer tissues and ANTTs	SOX4, and MMP-2/9	-	miR129-3p inhibits migration and invasion in RCC.	(86)
miR-99a	40 paired cancer tissues and ANTTs	mTOR	mTOR pathway	miR-99a inhibits tumorigenicity and tumor growth, and promotes G1-phase cell cycle arrest.	(87)
miR-203	24 paired cancer tissues and ANTTs	HOTAIR	PTEN pathway	miR-203 up-regulation reduces cell proliferation, migration, and invasion and induces apoptosis and cell-cycle arrest.	(88)
miR-145	15 paired cancer tissues and ANTTs	ADAM17	-	miR-145 suppresses proliferation and promotes cell apoptosis in RCC.	(89)
miR-22	68 paired cancer tissues and ANTTs	PTEN	Ras/mitogen-activated protein kinase pathway	miR-22 inhibits cell proliferation, migration and invasion.	(90)
miR-217	86 paired cancer tissues and ANTTs	HOTAIR, HIF-1α	HIF-1α/AXL signaling	miR-217 reduces proliferation, migratio, and EMT and increases apoptosis	(91)
miR-122-5p and miR-206	Serum samples from 68 ccRCC, 47 BRT, and 28 healthy controls	-	-	Serum expression levels of miR-122-5p and miR-206 are biomarkers for patients with ccRCC.	(92)
miR-199a-5p	9 paired cancer tissues and ANTTs	TGFBR1 and JunB	-	miR-199a-5p reduces invasion of ccRCC cells.	(93)
miR-10b	9 paired cancer tissues and ANTTs	-	-	miR-10b inhibits cell proliferation, invasive ability and migration, and induces cell cycle arrest.	(94)
miR‐30c	32 paired cancer tissues and ANTTs	Slug	-	miR‐30c suppresses EMT.	(95)
miR‐372	30 paired cancer tissues and ANTTs	IGF 2BP 1	-	miR‐372 as a tumor suppressor inhibits tumor progression, cell proliferation, cell invasion.	(96)
miR-186	20 paired cancer tissues and ANTTs	SENP1	NF-κB signaling pathway	miR-186 Suppresses cell Proliferation and invasion, and induces apoptosis.	(97)
miR-126	264 samples from primary ccRCC and 40 paired samples from cRCC patients	EGFL7, PIK3CD, VEGFA, and PIK3R2	HIF-1, VEGF, mTOR, and PI3K–Akt signaling pathways	miR-126 reduced cell proliferation and migration in RCC cells.	(98)
miR-10b	262 paired cancer tissues and ANTTs	PDGFB, ETS1, GRB2, PIK3CA, PIK3R3, CRK, BCL2 and MDM2	MAPK, Wnt and p53 signaling pathways	miR-10b has prognostic significance in ccRCC and its overexpression is associated with PDF and OS.	(84)
miR‐10a‐5p, ‐miR-10b‐5p	156 nephrectomy and 46 kidney biopsy specimens	-	-	Levels of miR‐10a‐5p, ‐10b‐5p may be biomarkers for ccRCC and they were correlated with cancer‐specific survival.	(44)
miR-182-5p	53 paired cancer tissues and ANTTs	MALAT-1	apoptotic pathways	miR-182-5p inhibits tumorigenicity and enhances apoptosis.	(99)
miR-144-3p	120 paired cancer tissues and ANTTs	MAP3K8	MAP3K8 pathway	miR-144-3p suppresses EMT, viability and metastasis.	(100)
MicroRNA-138	67 paired cancer tissues and ANTTs	SOX4		MiR-138 inhibits EMT, tumor growth, cell proliferation, migration and invasion.	(101)
miR-192 and miR-194	59 normal kidney and 54 tumor specimens; and 38 paired samples from cancer tissues and ANTTs	-	-	Two-miRNA combination is a potential predictor of renal cancer in patients.	(45)
miR‐124	30 paired cancer tissues and ANTTs	HOTAIR	-	miR‐124 inhibits RCC cell proliferation and metastasis.	(62)
miR-149-5p	16 paired cancer tissues and ANTTs	FOXM1	-	miR-149-5p suppresses Cell Migration and Invasion through Targeting FOXM1.	(102)
miR‐194	234 paired cancer tissues and ANTTs	HIF1A, MDM2,PIK3R2, MAPK1, IGF1R,BCL2, ITGB1, and CRK	HIF‐hypoxia pathway, VEGF, mTOR, Wnt, TGF‐beta, and MAPK signaling pathways	miR‐194 is a biomarker for prognosis in ccRCC.	(103)
miR-429	187 paired cancer tissues and ANTTs	E-cadheri	-	miR-429 inhibits cellular migration and cell motility.	(104)
miR-199a	150 paired cancer tissues and ANTTs	ROCK1		MiR-199a inhibits cell proliferation, migration and invasion.	(105)
miR-106a-5p	30 paired cancer tissues and ANTTs	PAK5	-	miR-106a-5p inhibits RCC progression and metastasis via PAK5.	(106)
miR-129-2	48 paired samples from cancer tissues and ANTTs	NKIRAS1 RARB(2),, CHL1 and RHOA	-	MIR-129-2 suppresses ccRCC progression.	(50)
miR-28-5p and miR-378	Serum from 107 RCC patients and 107 controls	-	-	These miRNAs may be diagnostic biomarker for RCC.	(51)
miR-30a-5p	249 cancer tissues and 71 matched normal samples	GRP78	miR-30a-5p/GRP78 signaling pathway	miR-30a-5p suppresses the cell growth and induces apoptosis in RCC.	(107)
miR-28-5p	33 paired cancer tissues and ANTTs	RAP1B	p38 and Erk1/2 pathways	miR-28-5p suppresses the tumorigenesis, cell proliferation, cell migration, and invasion.	(108)
miR-30e-3p	8 paired cancer tissues and ANTTs	Snail1		miR-30e-3p reduces cell invasion and migration.	(107)
miR-492	6 paired cancer tissues and ANTTs	-	-	miR-492 induces apoptosis and suppresses cell proliferation and invasion.	(109)
miR-137	45 paired cancer tissues and ANTTs	RLIP76	-	miR-137 inhibits cell growth and metastasis, and induces apoptosis.	(110)
miR-144	40 paired cancer tissues and ANTTs	MTOR	PI3K/AKT signaling pathway	miR-144 inhibits cell Proliferation and cell viability and promotes cell cycle arrest.	(111)
miR-34a, miR-200c and miR-141	paired serum samples from 30 patients	-	-	These miRNAs may be useful as diagnostic biomarkers.	(58)
miR-203	90 paired cancer tissues and ANTTs	FGF2		miR-203 inhibits cell proliferation, migration and invasion of RCC via inhibiting of FGF2.	(112)
hsa-miR-101	15 paired cancer tissues and ANTTs	TIGAR	-	hsa-miR-101 induces glycolysis and cell proliferation.	(113)
miR-137	50 paired cancer tissues and ANTTs	PI3K, p-AKT	PI3 K/AKT signaling pathway	miR-137 decreases cell proliferation, migratoin and invasion, and induces cell apoptosis.	(114)
miR-451	51 paired cancer tissues and ANTTs	PSMB8	inflammation pathway	miR-451 promotes cell apoptosis and suppresses cell proliferation and growth of RCC.	(115)
miR-497	86 paired cancer tissues and ANTTs	-	-	miR-497 reduces cell proliferation, migration and invasion of RCC.	(116)
miR-375	27 paired cancer tissues and ANTTs	YWHAZ	-	miR-375 inhibits cell proliferation, migration, and invasion.	(117)
miR-451	-	ATF-2	-	miR-451 enhanced drug resistance and cell apoptosis, and reduced cell viability.	(118)
miR-381	60 paired cancer tissues and ANTTs	-	-	miR-381 enhances cell apoptosis, and inhibits cell proliferation and chemoresistance.	(119)
miR-124	-	FZD5, P-gp	Wnt signaling pathway	miR-124 promotes cell apoptosis, and inhibits chemoresistance.	(120)

ANNTs, adjacent non-tumoral tissues.

### Diagnostic/Prognostic Value of miRNAs in RCC

Diagnostic and prognostic values of several miRNAs have been appraised in tissue samples, urine, or peripheral blood of RCC patients. A previous meta-analysis of available literature about miRNA signature in RCC tissues and their matching non-cancerous tissues has shown elevated levels of miR-21 and miR-210, while decreased levels of miR-141, miR-200c, and miR-429. Altered expressions of these miRNAs have been related with poor cancer-specific survival after tumor excision. Expression profile of these miRNA has been shown to be a suitable prognostic and predictive method for appraisal of survival of RCC patients particularly those with CCC (121).

An important application of miRNAs in the diagnostic process of RCC has been provided by their presence in the circulation of patients and their potential in liquid biopsy. Tusong et al. have reported over-expression of miR-21 and miR-106a in the serum samples of RCC patients compared with normal control samples. Notably, serum levels of these miRNAs have been decreased in patients a month after surgery suggesting their appropriateness as biomarkers for RCC (122). Wang et al. have reported consistent down-regulation of miR-200a in serum samples of patients with this kind of cancer, particularly in patients with stage I disease. Notably, level of this miRNA was commonly decreased in urine specimens of patients as well (123). A comprehensive assessment of miRNA profile in plasma specimens of ccRCC patients and healthy subjects has revealed the correlation between circulating miRNA signature and ccRCC stage. miRNA profiles were remarkably different between stage III/IV sections and both controls and early stage samples. Plasma levels of miR‐150 were considerably correlated with patients’ survival (124). A large-scale detection of formerly unannotated miRNA sequences in human renal specimens has led to identification of several miRNAs being dysregulated in ccRCC tumors and linked with poor survival of patients (125). Finally, experiments in a transgenic model of Xp11 RCC have shown higher amounts of miR‐204‐5p in urinary exosomes compared with control animals. Expression of this miRNA was also elevated in primary RCC cell lines created from transgenic mice indicating its role as a diagnostic marker for Xp11 tRCC (126).

**Table 3** gives a brief record of studies which reported the diagnostic/prognostic role of miRNAs in RCC.

**Table 3:** Diagnostic/prognostic role of miRNAs in renal cancer.

Samples	Area under curve	Sensitivity	Specificity	Kaplan-Meier analysis	Univariate cox regression	Multivariate cox regression	Reference
96 paired cancer tissues and ANTTs	0.675 for miRNA-135a-5p	45.5%	81.1%	Patients with lower expression of miRNA-135a-5p have higher metachronous metastasis.	Tumor necrosis, pT stage, Fuhrman grade, vascular invasion and lower miRNA-135a-5p levels were correlated with metachronous metastasis.	-	(80)
30 paired cancer tissues and ANTTs	0.905 for miR-720	80%	100%	Low expression of miR-720 indicated higher OS.	-	-	(32)
87 paired cancer tissues and ANTTs	-	–	–	Higher level of miR-124-3p was associated with better OS. Higher level of miR-200c-3p was associated with lower DFS and OS.	-	-	(82)
75 tumor tissues and 40 normal tissues	-	–	–	Higher miR‐766–3p levels were associated with better 5‐year OS.	A lower miR‐766–3p expression, a higher tumor size and a higher clinical T stage were associated with OS.	A lower miR‐766–3p expression was correlated with OS.	(84)
40 paired cancer tissues and ANTTs And 516 ccRCC patients from the TCGA database	-	–	–	Low miR-30a-5p expression was associated with short OS.	-	miR-30a-50p may be a prognostic marker in ccRCC patients.	(85)
69 paired cancer tissues and ANTTs	0.735 for miR-129-3p	75.9 %	62.1 %	miR-129-3p expression levels were associated with OS and DFS.	-	-	(86)
40 paired cancer tissues and ANTTs	-	–	–	Lower miR-99a expression level was correlated with decreased OS of RCC patients.	-	-	(87)
Plasma samples from 54 RCC patients and 50 healthy individuals	0.70 for miR-210, 0.62 for miR-221, 0.61 for miR-1233	60.9% for miR-210, 71.4% for miR-221, 39.1% for miR-1233	73.1% for miR-210, 65% for miR-221, 92.6% for miR-1233	Patients with higher levels of miR-210 and miR-1233 display a significantly lower cancer-specific survival.	-	-	(33)
148 ccRCC tissue samples along with 60 ANTTs				Patients with high miR‐122 levels display a significantly lower metastasis‐free survival rates than those with low miR‐122 levels.	High miR‐122 level is a poor prognostic factor for metastasis	High miR‐122 is an independent prognostic factor from gender, age, BMI, overall TNM, tumor size, grade and staging.	(34)
276 paired cancer tissues and ANTTs	-	–	–	patients with high miR‐125b expression had a poorer survival rate	High miR‐125b level was associated with shorter RFS.	Fuhrman grade, T stage and miR‐125b levels are independent prognostic factors for RFS.	(35)
Serum samples from 195 RCC patient and 100 healthy persons	0.85 for combination of miR-378 and miR-210	80% for combination of miR-378 and miR-210	78% for combination of miR-378 and miR-210	There were correlations between high serum miR-378 expression and clinical stage, and between miR-378 expression and DFS.	-	-	(36)
Serum samples from 68 ccRCC, 47 BRT, and 28 healthy controls	0.733 for the combination of miR-122-5p and miR-206	83.8% for the combination of miR-122-5p and miR-206	57.1% for the combination of miR-122-5p and miR-206	miR-122-5p and miR-206 expressions were associated with patients’ survival.	Increased miR-122-5p and miR-206 serum levels were associated with lower progression-free, cancer-specific, and OS.	miR-206 expression in serum has independent prognostic value in RCC.	(92)
40 paired cancer tissues and ANTTs	-	–	–	miR-203a was negatively correlated with outcome of RCC patients.	High miR-203a level was associated with higher pathological stage and shorter OS after radical nephrectomy.	High miR-203a level in RCC tissues suggests risk of RCC recurrence.	(39)
264 paired samples from primary ccRCC and 20 paired samples from metastatic ccRCC				miR-126 positivity was correlated with significantly higher DFS and OS.	Higher miR-126 expression associated with higher DFS and OS.		(53)
57 paired cancer tissues and ANTTs	-	–	–	miR-221 up-regulation was correlated with a poor PFS.	-	-	(43)
262 paired cancer tissues and ANTTs				Patients with higher miR-10b have longer DFS and OS compared with patients with lower miR-10b.	Patients with overexpressed miR-10b have higher DFS and OS.	Tumors with high miR-10b were associated with high DFS in comparison with tumors with low miR-10b.	(84)
156 nephrectomy and 46 kidney biopsy specimens	0.895 for combination of miR‐10a‐5p, miR‐10b‐5p, and miR‐223‐3p	86.7% for combination of miR‐10a‐5p, miR‐10b‐5p, and miR‐223‐3p	75% for combination of miR‐10a‐5p, miR‐10b‐5p, and miR‐223‐3p	Overexpression of miR‐10a‐5p and miR‐10b‐5p and down-regulation of miR‐223‐3p were significantly correlated with survival.	High grade, high stage, lower BMI, low miR‐10a‐5p, low miR‐10b‐5p, and high miR‐223‐3p expression were associated with death.	Stage, BMI, miR‐10a‐5p, and miR‐223‐3p expression were independent predictors of death.	(44)
53 paired cancer tissues and ANTTs	0.954 for miR-182-5p	90% for miR-182-5p	97% for miR-182-5p	-	Down-regulation of miR-182-5p was associated with an increase in Fuhrman grade.	-	(99)
120 paired cancer tissues and ANTTs	-	–	–	Low expression of miR-144-3p was significantly correlated with poor survival in RCC patients.	-	-	(100)
67 paired cancer tissues and ANTTs	-	–	–	Patients with lower miR-138 had worse OS and DFS than those with higher miR-138.	-	-	(101)
59 normal kidney and 54 tumor specimens; and 38 paired samples from cancer tissues and ANTTs	-	80% for combination of miR-21 and miR-194	97.5% for combination of miR-21 and miR-194	Lower expression of miRNA combinations (miR-21+194 miR-21+142-5p+194); was significantly associated with higher risk for metastasis.	miR-21 and miR-142-5p positively and miR-194 negatively associated with metastasis miRNA combinations (miR-21+194 and miR-21+142-5p+194). negatively associated with metastasis.	The miRNA combinations (miR-21+194; miR-21+142-5p+194) predicted metastasis.	(45)
234 paired cancer tissues and ANTTs				Higher expression of miR‐194 significantly associated with longer DFS and OS compared to lower expression levels.	miR‐194‐positive patients had longer DFS and OS.	miR‐194‐positive patients significantly associated with longer DFS and OS compared to those who are miR‐194‐negative	(103)
20 paired cancer tissues and ANTTs	-	–	–	High expression of miR-106b-5p associated with poor OS.	-	-	(49)
187 paired cancer tissues and ANTTs	-	–	–	Higher expression levels of miR-429 were correlated with longer DFS and OS.	-	-	(104)
123 paired cancer tissues and ANTTs	0.799 for hsa-miR-27b, 0.793 for hsa-miR-23b and 0.800 for hsa-miR-628-5p	–	–	Patients with overexpression of miR-628-5p and miR-27b have higher survival.			(49)
284 (264 primary and 20 metastatic ccRCC) paired cancer tissues and ANTTs	-	–	–	Patients with higher miR-210 expression had significantly lower DFS and OS.	-	miR-210 was not an independent prognostic marker for survival.	(53)
249 cancer tissues and 71 ANTTs	-	–	–	Down-regulation of miR-30a-5p and up-regulation of GRP78 were associated with shorter OS.	miR-30a-5p, TNM stage and grade, were independent prognostic factors for patients’ survival.	miR-30a-5p, TNM stage and grade were independent prognostic factors for patients’ survival.	(107)
45 paired cancer tissues and ANTTs	-	–	–	Up-regulation of miR-29b was significantly correlated with TNM stage and OS.	-	-	(59)
90 paired cancer tissues and ANTTs	-	–	–	Low level of miR-203 was associated with shorter OS.	OS of ccRCC patients was correlated with miR-203 expression level, tumor stage, lymph node metastasis, and histological grade.	Level of miR-203, tumor stage, lymph node metastasis, and histological grade were independent prognostic factors for OS.	(112)
15 paired cancer tissues and ANTTs	-	–	–	Down-regulation of miR-210-3p and up-regulation of TWIST1were correlated with poorer OS and DFS.	-	-	(60)
Serum samples from 82 ccRCC patients and 80 healthy controls	0.69 for miR-210 0.82 for miR-1233	70% for miR-210 81% for miR-1233	62.2% for miR-210 76% for miR-1233	-	-	-	(61)
51 paired cancer tissues and ANTTs	-	–	–	Down-regulation of miR-451 was associated with poor survival.	-	-	(115)
42 paired cancer tissues and ANTTs	-	–	–	High expression of miR-18a-5p was associated with poor survival.	Patients with higher miR-18a-5p expression had lower OS compared to patients with lower miR-18a-5p expression.	Down-regulation of miR-18a-5p was associated with better survival.	(62)
86 paired cancer tissues and ANTTs				Down-regulation of miR-497 was correlated with poor OS.	The OS of ccRCC patients was correlated with miR-497 expression, histological grade, tumor stage, and lymph node metastases.	miR-497 expression, tumor stage, histological grade, and lymph node metastases were correlated with OS.	(116)
516 tumor samples and 71 ANTTs	-	–	–	Up-regulation of miR-301a was associated with poor OS.	miR-301a is an independent prognostic marker for RCC patients.	miR-301a is an independent prognostic marker for RCC patients.	(22)
60 paired cancer tissues and ANTTs	-	–	–	Low expression of miR-206 was associated with poor OS.	-	miR-206, CDK6, TNM stage and lymph node metastasis as independent prognostic factors.	(73)
67 paired ccRCC tissues and ANTTs	-	–	–	Low level of miR‐143 was associated with poor OS.	-	-	(74)
18 clinical ccRCC tissue samples/5 patients with resistant to several tyrosine kinase inhibitor	-	–	–	Low expressions of miR‐101‐5p and miR‐101‐3p were correlated with high pathological grade, poor DFS and OS.	-	-	(127)
PRCC (292 tumor tissues and 34 normal) and ccRCC (545 tumor tissues and 71 normal), from TCGA database	-	–	–	High expression of miR-1293 was associated with poor OS.	-	-	(25)
90 primary ccRCC tissues and 90 ANTTs	-	–	–	High expression of miR-671-5p was associated with poor OS. .	miR-671-5p expression was an independent prognostic factor for OS.	miR-671-5p expression was an independent prognostic factor for OS.	(29)
114 paired ccRCC tissues and ANTTs and urine samples	-	–	–	High expression of miR-592 was associated with poor OS.	-	Expression of miR-592 and TNM stage were correlated with OS.	(31)
480 paired ccRCC tissues and ANTTs and urine samples	-	–	–	High expression of miR-22 was associated with poor OS.	-	-	(23)

ANNTs adjacent non-tumoral tissues OS overall survival RFS, relapse-free survival; DFS, disease-free survival.

### The Role of miRNAs in Determination of Response of RCC Patients to Treatment Modalities

Expression profile of a number of miRNAs correlates with response of RCC cells to chemotherapeutic agents. For instance, miR-381 has been shown to improve response of RCC cells to 5-fluorouracil through targeting WEE1 and enhancing activity of cyclin-dependent kinase 2 (128). Expression of miR-451 has been elevated in low multi-drug resistant (MDR);,;, cell line compared with the high MDR cell line. This miRNA has been shown to target ATF-2 and suppress its expression. Up-regulation of miR-451 has increased drug resistance, while its silencing improved response to chemotherapeutic agents (118). In the clinical settings, serum levels of miR-183 have been shown to predict response of RCC patients to cytotoxic effects of natural killer cells (129), implying the importance of miRNAs in immunotherapeutic options. A genome-wide miRNA profiling in RCC patients who received sunitinib showed lower levels of miR-141 in tumor samples of poor responders compared with good responders (81). Therefore, miRNAs modulate response of RCC patients to a wide range of treatment modalities. **Table 4** summarizes the impact of miRNAs in resistance to therapeutic modalities in RCC.

**Table 4:** Role of miRNAs in chemoresistance in RCC.

Response to chemotherapeutic drug	miRNA	Reference
Cisplatin and paclitaxel sensitivity	miR-381	(119)
paclitaxel sensitivity	miR-381	(119)
Sunitinib resistance	miR-144-3p	(57)
adriamycin resistance	miR-451	(118)
oxaliplatin resistance	miR-489-3p and miR-630	(63)
vinblastine and doxorubicin sensitivity	miR-124	(120)
paclitaxel, 5-fluorouracil, oxaliplatin, and dovitinib resistance	miR-21	(64)

## Discussion

The oncogenic function of numerous miRNAs has been proved in RCC cells These oncomiRs have been shown to enhance cell proliferation and invasive features of RCC cells whilst decreasing apoptosis Notably some tumor suppressor genes such PTEN APC and MEG3 have been identified as targets of oncomiRs such as miR-301a miR-193a-3p miR-22 miR-671-5p, and miR-7, indicating a possible mechanism for their participation in the pathogenesis of RCC. Instead, tumor suppressor miRNAs which are down-regulated in RCC cells have potential roles in the activation of apoptotic pathways and arrestment of cell cycle transition. A number of these miRNAs target EMT-associated genes such as ZEB1, Slug, HOTAIR, and HIF-1α. Thus, their down-regulation is associated with the enhancement of EMT program. miRNAs are regarded as potential markers of different malignancies including RCC. These transcripts regulate several cancer-related cellular functions such as apoptosis, survival, migration and angiogenesis. Therefore, several miRNAs have similar functions and expression profiles in diverse cancers. Although aberrant expression of miRNAs in cancer patients is a useful tool for follow-up of patients, identification of tissue-specific pattern of their expression is necessary to differentiate between different cancers originating from a certain body system. In spite of extensive efforts for biomarker discovery, there is no consensus on miRNA panels that are specific for a certain type of cancer. A previous study has reported up-regulation of miR-28, miR-185, miR-27, and let-7f-2 in RCC samples, whereas expression of a different set of miRNAs including miR-223, miR-26b, miR-221, and miR-103-1was increased in bladder cancer samples. Based on these results, authors suggested the potential of miRNAs in differentiating between these two types of cancers (20). However, others have reported over-expression of bladder cancer-related miRNAs such as miR-223 and miR-221 in RCC samples (33, 130) casting doubt on the possibility of identification of tissue-specific miRNA signature in different cancers. Studies which appraised the biomarker role of miRNAs in RCC suffer from small sample size, inclusion of samples from diverse clinical stages and histologic subclasses as well as benign kidney lesions and validation in independent samples. Possibly, the most important limitation of miRNAs as diagnostic markers is their inability for differentiation between malignancies with diverse origins. Based on this limitation, they cannot be used for primary diagnosis of cancer but for patients’ follow-up. Another possible application of miRNAs in the RCC patients rises from their importance in the determination of patients’ response to chemotherapy. Therefore, a prior identification of miRNA profile in the biopsy samples might facilitate selection of the most appropriate therapeutic regimen in a personalized manner. Moreover, targeted suppression of certain miRNAs is a possible modality to enhance response of patients to chemotherapy. miR-21 represents a promising candidate in this regard, since it has been shown to be over-expressed in RCC samples in independent studies and its silencing has enhance response to multiple anti-cancer drugs such as paclitaxel, 5-fluorouracil, oxaliplatin, and dovitinib. Yet, miRNA-based therapies face a number of challenges such as design of specific formulations to avoid off-target effects and low efficacy of delivery methods (131).

Comparison of miRNA levels in serum and tissue samples of RCC patients and healthy subjects has led to identification of several dysregulated miRNAs in serum samples. Yet, only a fraction of these miRNAs have been dysregulated in tissue samples, implying that a minor portion of circulating miRNAs have been originated from the tumor tissues (21). Therefore, future studies are needed to explore the source of circulating miRNAs in RCC patients. Based on the results of recent investigations, both serum and urine samples of patients with RCC might be used as sources for discovery of miRNA levels, facilitating conduction of non-invasive methods for RCC diagnosis.

miRNA signature can be used for classification of RCC subtypes. The miRNA-based classification system developed by Youssef et al. could discriminate different subtypes of RCC such as clear cell, papillary, oncocytoma, and chromophobe RCC with sensitivity values between 97% and 100% (132). Moreover, miR-15a has been shown to have distinct expression pattern between RCC and oncocytoma being up-regulated in the former, yet down-regulated in the latter. Expression of this miRNA was similarly up-regulated in chromophobe carcinoma, while in the papillary RCC samples miR-15a expression was not such over-expressed. Over-expression of miR-15a was also detectable in urine samples of RCC patients. However, miR-15a was almost untraceable in oncocytoma, other tumors, and inflammatory disorders of the urinary tract (133). These results indicate the possibility of substitution of histopathological classification methods by molecular methods. The clinical implications of these findings should be confirmed in larger samples of patients.

Taken together, miRNAs participate in the pathogenesis of RCC and response of patients to diverse therapeutic modalities. Moreover, as they are traceable in circulation and urine samples of patients, they can be used as biomarkers for this kind of cancer. However, at the present time, there is no miRNA that can be widely applied as biomarker or treatment target in the clinical setting. This is partly because of the heterogeneous pattern of expression of miRNAs in RCC samples and circulation of patients. This research filed lacks comprehensive assessment of miRNA profiles in large cohorts of RCC patients. Therefore, future studies with these features are expected to facilitate design of suitable diagnostic panels containing miRNAs.

## Author Contributions

MT and SG-F wrote the draft and revised it. ZS-F and WB designed the tables and study, and performed the data collection. All authors contributed to the article and approved the submitted version.

## Conflict of Interest

The authors declare that the research was conducted in the absence of any commercial or financial relationships that could be construed as a potential conflict of interest.

## References

[1] Bray F Ferlay J Soerjomataram I Siegel RL Torre LA Jemal A Global cancer statistics 2018: GLOBOCAN estimates of incidence and mortality worldwide for 36 cancers in 185 countries. CA: Cancer J Clin (2018) 68:394–424. 10.3322/caac.21492 30207593

[2] Capitanio U Bensalah K Bex A Boorjian SA Bray F Coleman J Epidemiology of Renal Cell Carcinoma. Eur Urol (2019) 75(1):74–84. 10.1016/j.eururo.2018.08.036 30243799 PMC8397918

[3] Rossi SH Klatte T Usher-Smith J Stewart GD Epidemiology and screening for renal cancer. World J Urol (2018) 36(9):1341–53. 10.1007/s00345-018-2286-7 PMC6105141 29610964

[4] Morrissey JJ Mobley J Figenshau RS Vetter J Bhayani S Kharasch ED Urine aquaporin 1 and perilipin 2 differentiate renal carcinomas from other imaged renal masses and bladder and prostate cancer. Mayo Clin Proc (2015) 90(1):35–42. 10.1016/j.mayocp.2014.10.005 25572193 PMC4317334

[5] Wang H Peng R Wang J Qin Z Xue L Circulating microRNAs as potential cancer biomarkers: the advantage and disadvantage. Clin Epigenet (2018) 10(1):1–10. 10.1186/s13148-018-0492-1 PMC5913875 29713393

[6] Bracken CP Scott HS Goodall GJ. A network-biology perspective of microRNA function and dysfunction in cancer. Nat Rev Genet (2016) 17(12):719–32. 10.1038/nrg.2016.134 27795564

[7] Ha M Kim VN Regulation of microRNA biogenesis. Nat Rev Mol Cell Biol (2014) 15(8):509–24. 10.1038/nrm3838 25027649

[8] Kehl T Backes C Kern F Fehlmann T Ludwig N Meese E About miRNAs, miRNA seeds, target genes and target pathways. Oncotarget (2017) 8(63):107167–75. 10.18632/oncotarget.22363 PMC5739805 29291020

[9] Baek D Villén J Shin C Camargo FD Gygi SP Bartel DP The impact of microRNAs on protein output. Nature (2008) 455(7209):64–71. 10.1038/nature07242 18668037 PMC2745094

[10] Bushati N Cohen SM microRNA functions. Annu Rev Cell Dev Biol (2007) 23:175–205. 10.1146/annurev.cellbio.23.090506.123406 17506695

[11] Jansson MD Lund AH MicroRNA and cancer. Mol Oncol (2012) 6(6):590–610. 10.1016/j.molonc.2012.09.006 23102669 PMC5528350

[12] Peng Y Croce CM The role of MicroRNAs in human cancer. Signal Transduct Target Ther (2016) 1(1):1–9. 10.1038/sigtrans.2015.4 PMC5661652 29263891

[13] Calin GA Dumitru CD Shimizu M Bichi R Zupo S Noch E Frequent deletions and down-regulation of micro-RNA genes miR15 and miR16 at 13q14 in chronic lymphocytic leukemia. Proc Natl Acad Sci (2002) 99(24):15524–9. 10.1073/pnas.242606799 PMC137750 12434020

[14] Calin GA Croce C MicroRNAs and chromosomal abnormalities in cancer cells. Oncogene (2006) 25(46):6202–10. 10.1038/sj.onc.1209910 17028600

[15] Zhang L Huang J Yang N Greshock J Megraw MS Giannakakis A microRNAs exhibit high frequency genomic alterations in human cancer. Proc Natl Acad Sci (2006) 103(24):9136–41. 10.1073/pnas.0508889103 PMC1474008 16754881

[16] O’Donnell KA Wentzel EA Zeller KI Dang CV Mendell JT c-Myc-regulated microRNAs modulate E2F1 expression. Nature (2005) 435(7043):839–43. 10.1038/nature03677 15944709

[17] Chang T-C Yu D Lee Y-S Wentzel EA Arking DE West KM Widespread microRNA repression by Myc contributes to tumorigenesis. Nat Genet (2008) 40(1):43–50. 10.1038/ng.2007.30 18066065 PMC2628762

[18] Mytsyk Y Dosenko V Skrzypczyk MA Borys Y Diychuk Y Kucher A Potential clinical applications of microRNAs as biomarkers for renal cell carcinoma. Cent Eur J Urol (2018) 71(3):295–303. 10.5173/ceju.2018.1618 PMC6202627 30386650

[19] Li J Huang J-H Qu Q-H Xia Q Wang D-S Jin L Evaluating the microRNA-target gene regulatory network in renal cell carcinomas, identification for potential biomarkers and critical pathways. Int J Clin Exp Med (2015) 8(5):7209–19. PMC4509205 26221260

[20] Gottardo F Liu CG Ferracin M Calin GA Fassan M Bassi P Micro-RNA profiling in kidney and bladder cancers. Urol Oncol (2007) 25(5):387–92. 10.1016/j.urolonc.2007.01.019 17826655

[21] Wulfken LM Moritz R Ohlmann C Holdenrieder S Jung V Becker F MicroRNAs in renal cell carcinoma: diagnostic implications of serum miR-1233 levels. PloS One (2011) 6(9):e25787. 10.1371/journal.pone.0025787 21984948 PMC3184173

[22] Li J Jiang D Zhang Q Peng S Liao G Yang X MiR-301a Promotes Cell Proliferation by Repressing PTEN in Renal Cell Carcinoma. Cancer Manag Res (2020) 12:4309–20. 10.2147/CMAR.S253533 PMC7294045 32606927

[23] Gong X Zhao H Saar M Peehl DM Brooks JD miR-22 Regulates Invasion, Gene Expression and Predicts Overall Survival in Patients with Clear Cell Renal Cell Carcinoma. Kidney Cancer (Clifton Va) (2019) 3(2):119–32. 10.3233/KCA-190051 PMC6839454 31763513

[24] Liu L Li Y Liu S Duan Q Chen L Wu T Downregulation of miR-193a-3p inhibits cell growth and migration in renal cell carcinoma by targeting PTEN. Tumor Biol (2017) 39(6):1010428317711951. 10.1177/1010428317711951 28639901

[25] Liu XL Pan WG Li KL Mao YJ Liu SD Zhang RM miR-1293 Suppresses Tumor Malignancy by Targeting Hydrocyanic Oxidase 2: Therapeutic Potential of a miR-1293/Hydrocyanic Oxidase 2 Axis in Renal Cell Carcinoma. Cancer Biother Radiopharm (2020) 35(5):377–86. 10.1089/cbr.2019.2957 31971830

[26] Wang J Wang C Li Q Guo C Sun W Zhao D miR-429-CRKL axis regulates clear cell renal cell carcinoma malignant progression through SOS1/MEK/ERK/MMP2/MMP9 pathway. BioMed Pharmacother (2020) 127:110215. 10.1016/j.biopha.2020.110215 32413671

[27] Zeng R Huang J Sun Y Luo J Cell proliferation is induced in renal cell carcinoma through miR-92a-3p upregulation by targeting FBXW7. Oncol Lett (2020) 19(4):3258–68. 10.3892/ol.2020.11443 PMC7074420 32256821

[28] Petrozza V Costantini M Tito C Giammusso LM Sorrentino V Cacciotti J Emerging role of secreted miR-210-3p as potential biomarker for clear cell Renal Cell Carcinoma metastasis. Cancer Biomark (2020) 27(2):181–8. 10.3233/CBM-190242 PMC12662287 31771042

[29] Chi XG Meng XX Ding DL Xuan XH Chen YZ Cai Q HMGA1-mediated miR-671-5p targets APC to promote metastasis of clear cell renal cell carcinoma through Wnt signaling. Neoplasma (2020) 67(1):46–53. 10.4149/neo_2019_190217N135 31686521

[30] Liu F Chen Y Chen B Liu C Xing J MiR-935 Promotes Clear Cell Renal Cell Carcinoma Migration and Invasion by Targeting IREB2. Cancer Manag Res (2019) 11:10891–900. 10.2147/CMAR.S232380 PMC6941696 31920398

[31] Lv X Shen J Guo Z Kong L Zhou G Ning H Aberrant Expression of miR-592 Is Associated with Prognosis and Progression of Renal Cell Carcinoma. Onco Targets Ther (2019) 12:11231–9. 10.2147/OTT.S227834 PMC6927226 31908489

[32] Bhat NS Colden M Dar AA Saini S Arora P Shahryari V MicroRNA-720 Regulates E-cadherin–αE-catenin Complex and Promotes Renal Cell Carcinoma. Mol Cancer Ther (2017) 16(12):2840–8. 10.1158/1535-7163.MCT-17-0400 PMC5893503 28802251

[33] Dias F Teixeira AL Ferreira M Adem B Bastos N Vieira J Plasmatic miR-210, miR-221 and miR-1233 profile: potential liquid biopsies candidates for renal cell carcinoma. Oncotarget (2017) 8(61):103315–26. 10.18632/oncotarget.21733 PMC5732730 29262564

[34] Fan Y Ma X Li H Gao Y Huang Q Zhang Y miR-122 promotes metastasis of clear-cell renal cell carcinoma by downregulating Dicer. Int J cancer (2018) 142(3):547–60. 10.1002/ijc.31050 28921581

[35] Fu Q Liu Z Pan D Zhang W Xu L Zhu Y Tumor miR-125b predicts recurrence and survival of patients with clear-cell renal cell carcinoma after surgical resection. Cancer Sci (2014) 105(11):1427–34. 10.1111/cas.12507 PMC4462383 25155155

[36] Fedorko M Stanik M Iliev R Redova-Lojova M Machackova T Svoboda M Combination of MiR-378 and MiR-210 Serum Levels Enables Sensitive Detection of Renal Cell Carcinoma. Int J Mol Sci (2015) 16(10):23382–9. 10.3390/ijms161023382 PMC4632704 26426010

[37] Fujii N Hirata H Ueno K Mori J Oka S Shimizu K Extracellular miR-224 as a prognostic marker for clear cell renal cell carcinoma. Oncotarget (2017) 8(66):109877–88. 10.18632/oncotarget.22436 PMC5746350 29299115

[38] He H Dai J Zhuo R Zhao J Wang H Sun F Study on the mechanism behind lncRNA MEG3 affecting clear cell renal cell carcinoma by regulating miR-7/RASL11B signaling. J Cell Physiol (2018) 233(12):9503–15. 10.1002/jcp.26849 29968912

[39] Hu G Lai P Liu M Xu L Guo Z Liu H miR-203a regulates proliferation, migration, and apoptosis by targeting glycogen synthase kinase-3β in human renal cell carcinoma. Tumor Biol (2014) 35(11):11443–53. 10.1007/s13277-014-2476-x 25123268

[40] Ji H Tian D Zhang B Zhang Y Yan D Wu S Overexpression of miR-155 in clear-cell renal cell carcinoma and its oncogenic effect through targeting FOXO3a. Exp Ther Med (2017) 13(5):2286–92. 10.3892/etm.2017.4263 PMC5443202 28565840

[41] Jin L Zhang Z Li Y He T Hu J Liu J miR-125b is associated with renal cell carcinoma cell migration, invasion and apoptosis. Oncol Lett (2017) 13(6):4512–20. 10.3892/ol.2017.5985 PMC5453059 28599452

[42] Jingushi K Kashiwagi Y Ueda Y Kitae K Hase H Nakata W High miR-122 expression promotes malignant phenotypes in ccRCC by targeting occludin. Int J Oncol (2017) 51(1):289–97. 10.3892/ijo.2017.4016 28534944

[43] Khella HWZ Butz H Ding Q Rotondo F Evans KR Kupchak P miR-221/222 Are Involved in Response to Sunitinib Treatment in Metastatic Renal Cell Carcinoma. Mol Ther J Am Soc Gene Ther (2015) 23(11):1748–58. 10.1038/mt.2015.129 PMC4817948 26201448

[44] Kowalik CG Palmer DA Sullivan TB Teebagy PA Dugan JM Libertino JA Profiling microRNA from nephrectomy and biopsy specimens: predictors of progression and survival in clear cell renal cell carcinoma. BJU Int (2017) 120(3):428–40. 10.1111/bju.13886 28432832

[45] Lokeshwar SD Talukder A Yates TJ Hennig MJP Garcia-Roig M Lahorewala SS Molecular Characterization of Renal Cell Carcinoma: A Potential Three-MicroRNA Prognostic Signature. Cancer Epidemiol Biomarkers Prevent (2018) 27(4):464–72. 10.1158/1055-9965.EPI-17-0700 29440068

[46] Pan Y Hu J Ma J Qi X Zhou H Miao X MiR-193a-3p and miR-224 mediate renal cell carcinoma progression by targeting alpha-2,3-sialyltransferase IV and the phosphatidylinositol 3 kinase/Akt pathway. Mol Carcinogenesis (2018) 57(8):1067–77. 10.1002/mc.22826 29667779

[47] Oliveira RdC Ivanovic RF Leite KRM Viana NI Pimenta RCA Junior JP Expression of micro-RNAs and genes related to angiogenesis in ccRCC and associations with tumor characteristics. BMC Urol (2017) 17(1):113. 10.1186/s12894-017-0306-3 29202733 PMC5715647

[48] Lu J Wei J-H Feng Z-H Chen Z-H Wang Y-Q Huang Y miR-106b-5p promotes renal cell carcinoma aggressiveness and stem-cell-like phenotype by activating Wnt/β-catenin signalling. Oncotarget (2017) 8(13):21461–71. 10.18632/oncotarget.15591 PMC5400598 28423523

[49] Puente J Laínez N Dueñas M Méndez-Vidal MJ Esteban E Castellano D Novel potential predictive markers of sunitinib outcomes in long-term responders versus primary refractory patients with metastatic clear-cell renal cell carcinoma. Oncotarget (2017) 8(18):30410–21. 10.18632/oncotarget.16494 PMC5444752 28423742

[50] Pronina IV Klimov EA Burdennyy AM Beresneva EV Fridman MV Ermilova VD Methylation of the genes for the microRNAs miR-129-2 and miR-9-1, changes in their expression, and activation of their potential target genes in clear cell renal cell carcinoma. Mol Biol (2017) 51(1):61–71. 10.1134/S0026893316060169 28251969

[51] Wang C Hu J Lu M Gu H Zhou X Chen X A panel of five serum miRNAs as a potential diagnostic tool for early-stage renal cell carcinoma. Sci Rep (2015) 5:7610–0. 10.1038/srep07610 PMC5154588 25556603

[52] Toraih EA Ibrahiem AT Fawzy MS Hussein MH Al-Qahtani SAM Shaalan AAM MicroRNA-34a: A Key Regulator in the Hallmarks of Renal Cell Carcinoma. Oxid Med Cell Longevity (2017) 2017:3269379–3269379. 10.1155/2017/3269379 PMC5632457 29104726

[53] Samaan S Khella HW Girgis A Scorilas A Lianidou E Gabril M miR-210 is a prognostic marker in clear cell renal cell carcinoma. J Mol Diagnostics JMD (2015) 17(2):136–44. 10.1016/j.jmoldx.2014.10.005 25555365

[54] Wang Z Qin C Zhang J Han Z Tao J Cao Q MiR-122 promotes renal cancer cell proliferation by targeting Sprouty2. Tumor Biol (2017) 39(2):1010428317691184. 10.1177/1010428317691184 28231730

[55] Wotschofsky Z Gummlich L Liep J Stephan C Kilic E Jung K Integrated microRNA and mRNA Signature Associated with the Transition from the Locally Confined to the Metastasized Clear Cell Renal Cell Carcinoma Exemplified by miR-146-5p. PloS One (2016) 11(2):e0148746–e0148746. 10.1371/journal.pone.0148746 26859141 PMC4747468

[56] Xiang W He J Huang C Chen L Tao D Wu X miR-106b-5p targets tumor suppressor gene SETD2 to inactive its function in clear cell renal cell carcinoma. Oncotarget (2015) 6(6):4066–79. 10.18632/oncotarget.2926 PMC4414173 25714014

[57] Xiao W Lou N Ruan H Bao L Xiong Z Yuan C Mir-144-3p Promotes Cell Proliferation, Metastasis, Sunitinib Resistance in Clear Cell Renal Cell Carcinoma by Downregulating ARID1A. Cell Physiol Biochem (2017) 43(6):2420–33. 10.1159/000484395 29073615

[58] Yadav S Khandelwal M Seth A Saini AK Dogra PN Sharma A Serum microRNA Expression Profiling: Potential Diagnostic Implications of a Panel of Serum microRNAs for Clear Cell Renal Cell Cancer. Urology (2017) 104:64–9. 10.1016/j.urology.2017.03.013 28336290

[59] Xu Y Zhu J Lei Z Wan L Zhu X Ye F Expression and functional role of miR-29b in renal cell carcinoma. Int J Clin Exp Pathol (2015) 8(11):14161–70. PMC4713515 26823729

[60] Yoshino H Yonemori M Miyamoto K Tatarano S Kofuji S Nohata N microRNA-210-3p depletion by CRISPR/Cas9 promoted tumorigenesis through revival of TWIST1 in renal cell carcinoma. Oncotarget (2017) 8(13):20881–94. 10.18632/oncotarget.14930 PMC5400553 28152509

[61] Xu Y Deng W Zhang W Long non-coding RNA TUG1 protects renal tubular epithelial cells against injury induced by lipopolysaccharide via regulating microRNA-223. Biomed Pharmacother (2018) 104:509–19. 10.1016/j.biopha.2018.05.069 29800915

[62] Zhou L Li Z Pan X Lai Y Quan J Zhao L Identification of miR-18a-5p as an oncogene and prognostic biomarker in RCC. Am J Trans Res (2018) 10(6):1874–86. PMC6038077 30018727

[63] Chen L Chen L Qin Z Lei J Ye S Zeng K Upregulation of miR-489-3p and miR-630 inhibits oxaliplatin uptake in renal cell carcinoma by targeting OCT2. Acta Pharm Sin B (2019) 9(5):1008–20. 10.1016/j.apsb.2019.01.002 PMC6804444 31649850

[64] Gaudelot K Gibier JB Pottier N Hémon B Van Seuningen I Glowacki F Targeting miR-21 decreases expression of multi-drug resistant genes and promotes chemosensitivity of renal carcinoma. Tumour Biol J Int Soc Oncodevelopmental Biol Med (2017) 39(7):1010428317707372. 10.1177/1010428317707372 28714373

[65] Chen J Gu Y Shen W MicroRNA-21 functions as an oncogene and promotes cell proliferation and invasion via TIMP3 in renal cancer. Eur Rev Med Pharmacol Sci (2017) 21(20):4566–76. 29131259

[66] Nakada C Matsuura K Tsukamoto Y Tanigawa M Yoshimoto T Narimatsu T Genome-wide microRNA expression profiling in renal cell carcinoma: significant down-regulation of miR-141 and miR-200c. J Pathol: A J Pathol Soc Great Britain Ireland (2008) 216(4):418–27. 10.1002/path.2437 18925646

[67] Onyshchenko KV Voitsitskyi TV Grygorenko VM Saidakova NO Pereta LV Onyschuk AP Expression of micro-RNA hsa-miR-30c-5p and hsa-miR-138-1 in renal cell carcinoma. Exp Oncol (2020) 42(2):115–9. 10.32471/exp-oncology.2312-8852.vol-42-no-2.14632 32602286

[68] Zhu H Wang S Shen H Zheng X Xu X SP1/AKT/FOXO3 Signaling Is Involved in miR-362-3p-Mediated Inhibition of Cell-Cycle Pathway and EMT Progression in Renal Cell Carcinoma. Front Cell Dev Biol (2020) 8:297. 10.3389/fcell.2020.00297 32432112 PMC7214730

[69] Li Y Guan B Liu J Zhang Z He S Zhan Y MicroRNA-200b is downregulated and suppresses metastasis by targeting LAMA4 in renal cell carcinoma. EBioMedicine (2019) 44:439–51. 10.1016/j.ebiom.2019.05.041 PMC6604878 31130475

[70] Xie Y Chen L Gao Y Ma X He W Zhang Y miR-363 suppresses the proliferation, migration and invasion of clear cell renal cell carcinoma by downregulating S1PR1. Cancer Cell Int (2020) 20:227. 10.1186/s12935-020-01313-9 32536815 PMC7288407

[71] Xu H Wu S Shen X Shi Z Wu D Yuan Y Methylation-mediated miR-214 regulates proliferation and drug sensitivity of renal cell carcinoma cells through targeting LIVIN. J Cell Mol Med (2020) 24(11):6410–25. 10.1111/jcmm.15287 PMC7294148 32395888

[72] Xu Y Ma Y Liu XL Gao SL miR−133b affects cell proliferation, invasion and chemosensitivity in renal cell carcinoma by inhibiting the ERK signaling pathway. Mol Med Rep (2020) 22(1):67–76. 10.3892/mmr.2020.11125 32377748 PMC7248518

[73] Guo Z Jia H Ge J MiR-206 suppresses proliferation and epithelial-mesenchymal transition of renal cell carcinoma by inhibiting CDK6 expression. Hum Cell (2020) 33(3):750–8. 10.1007/s13577-020-00355-5 32277426

[74] Xu B Wang C Wang YL Chen S-Q Wu J-P Zhu W-D miR-143 inhibits renal cell carcinoma cells metastatic potential by suppressing ABL2. Kaohsiung J Med Sci (2020) 36(8). 10.1002/kjm2.12207 PMC11896136 32196963

[75] Chen J Zhong Y Li L miR-124 and miR-203 synergistically inactivate EMT pathway via coregulation of ZEB2 in clear cell renal cell carcinoma (ccRCC). J Transl Med (2020) 18(1):69. 10.1186/s12967-020-02242-x 32046742 PMC7014595

[76] Yamada Y Nohata N Uchida A Kato M Arai T Moriya S Replisome genes regulation by antitumor miR-101-5p in clear cell renal cell carcinoma. Cancer Sci (2020) 111(4):1392–406. 10.1111/cas.14327 PMC7156888 31975570

[77] Xiao W Wang C Chen K Wang T Xing J Zhang X MiR-765 functions as a tumour suppressor and eliminates lipids in clear cell renal cell carcinoma by downregulating PLP2. EBioMedicine (2020) 51:102622. 10.1016/j.ebiom.2019.102622 31901870 PMC6948168

[78] Deng JH Zheng GY Li HZ Ji ZG MiR-212-5p inhibits the malignant behavior of clear cell renal cell carcinoma cells by targeting TBX15. Eur Rev Med Pharmacol Sci (2019) 23(24):10699–707. 10.26355/eurrev_201912_19770 31858538

[79] Gilyazova IR Klimentova EA Bulygin KV MicroRNA-200 family expression analysis in metastatic clear cell renal cell carcinoma patients. Cancer Gene Ther (2019). 10.1038/s41417-019-0149-z 31680118

[80] Shiomi E Sugai T Ishida K Osakabe M Tsuyukubo T Kato Y Analysis of Expression Patterns of MicroRNAs That Are Closely Associated With Renal Carcinogenesis. Front Oncol (2019) 9:431. 10.3389/fonc.2019.00431 31214494 PMC6555129

[81] Berkers J Govaere O Wolter P Beuselinck B Schöffski P Kempen LCv A Possible Role for MicroRNA-141 Down-Regulation in Sunitinib Resistant Metastatic Clear Cell Renal Cell Carcinoma Through Induction of Epithelial-to-Mesenchymal Transition and Hypoxia Resistance. J Urol (2013) 189(5):1930–8. 10.1016/j.juro.2012.11.133 23206420

[82] Butz H Szabó PM Khella HW Nofech-Mozes R Patocs A Yousef GM miRNA-target network reveals miR-124as a key miRNA contributing to clear cell renal cell carcinoma aggressive behaviour by targeting CAV1 and FLOT1. Oncotarget (2015) 6(14):12543–57. 10.18632/oncotarget.3815 PMC4494957 26002553

[83] Cao H Liu Z Wang R Zhang X Yi W Nie G miR-148a suppresses human renal cell carcinoma malignancy by targeting AKT2. Oncol Rep (2017) 37(1):147–54. 10.3892/or.2016.5257 27878305

[84] Khella HWZ Daniel N Youssef L Scorilas A Nofech-Mozes R Mirham L miR-10b is a prognostic marker in clear cell renal cell carcinoma. J Clin Pathol (2017) 70(10):854–9. 10.1136/jclinpath-2017-204341 28360191

[85] Chen Z Zhang J Zhang Z Feng Z Wei J Lu J The putative tumor suppressor microRNA-30a-5p modulates clear cell renal cell carcinoma aggressiveness through repression of ZEB2. Cell Death Disease (2017) 8(6):e2859–9. 10.1038/cddis.2017.252 PMC5520909 28569782

[86] Chen X Ruan A Wang X Han W Wang R Lou N miR-129-3p, as a diagnostic and prognostic biomarker for renal cell carcinoma, attenuates cell migration and invasion via downregulating multiple metastasis-related genes. J Cancer Res Clin Oncol (2014) 140(8):1295–304. 10.1007/s00432-014-1690-7 PMC11824026 24802708

[87] Cui L Zhou H Zhao H Zhou Y Xu R Xu X MicroRNA-99a induces G1-phase cell cycle arrest and suppresses tumorigenicity in renal cell carcinoma. BMC Cancer (2012) 12(1):546. 10.1186/1471-2407-12-546 23173671 PMC3518250

[88] Dasgupta P Kulkarni P Majid S Shahryari V Hashimoto Y Bhat NS MicroRNA-203 Inhibits Long Noncoding RNA HOTAIR and Regulates Tumorigenesis through Epithelial-to-mesenchymal Transition Pathway in Renal Cell Carcinoma. Mol Cancer Ther (2018) 17(5):1061–9. 10.1158/1535-7163.MCT-17-0925 PMC5932222 29440295

[89] Doberstein K Steinmeyer N Hartmetz A-K Eberhardt W Mittelbronn M Harter PN MicroRNA-145 Targets the Metalloprotease ADAM17 and Is Suppressed in Renal Cell Carcinoma Patients. Neoplasia (2013) 15(2):218–IN231. 10.1593/neo.121222 23441135 PMC3579323

[90] Fan W Huang J Xiao H Liang Z MicroRNA-22 is downregulated in clear cell renal cell carcinoma, and inhibits cell growth, migration and invasion by targeting PTEN. Mol Med Rep (2016) 13(6):4800–6. 10.3892/mmr.2016.5101 27082730

[91] Hong Q Li O Zheng W Xiao W-z Zhang L Wu D LncRNA HOTAIR regulates HIF-1α/AXL signaling through inhibition of miR-217 in renal cell carcinoma. Cell Death Disease (2017) 8(5):e2772–2. 10.1038/cddis.2017.181 PMC5520706 28492542

[92] Heinemann FG Tolkach Y Deng M Schmidt D Perner S Kristiansen G Serum miR-122-5p and miR-206 expression: non-invasive prognostic biomarkers for renal cell carcinoma. Clin Epigenetics (2018) 10(1):11. 10.1186/s13148-018-0444-9 29410711 PMC5781339

[93] He H Wang L Zhou W Zhang Z Wang L Xu S MicroRNA expression profiling in clear cell renal cell carcinoma: identification and functional validation of key miRNAs. PloS One (2015) 10(5):e0125672. 10.1371/journal.pone.0125672 25938468 PMC4418764

[94] He C Zhao X Jiang H Zhong Z Xu R Demethylation of miR-10b plays a suppressive role in ccRCC cells. Int J Clin Exp Pathol (2015) 8(9):10595–604. PMC4637584 26617769

[95] Huang J Yao X Zhang J Dong B Chen Q Xue W Hypoxia-induced downregulation of miR-30c promotes epithelial-mesenchymal transition in human renal cell carcinoma. Cancer Sci (2013) 104(12):1609–17. 10.1111/cas.12291 PMC7653518 24112779

[96] Huang X Huang M Kong L Li Y miR-372 suppresses tumour proliferation and invasion by targeting IGF2BP1 in renal cell carcinoma. Cell Proliferation (2015) 48(5):593–9. 10.1111/cpr.12207 PMC6496015 26332146

[97] Jiao D Wu M Ji L Liu F Liu Y MicroRNA-186 Suppresses Cell Proliferation and Metastasis Through Targeting Sentrin-Specific Protease 1 in Renal Cell Carcinoma. Oncol Res (2018) 26(2):249–59. 10.3727/096504017X14953948675430 PMC7844750 28550686

[98] Khella HW Scorilas A Mozes R Mirham L Lianidou E Krylov SN Low expression of miR-126 is a prognostic marker for metastatic clear cell renal cell carcinoma. Am J Pathol (2015) 185(3):693–703. 10.1016/j.ajpath.2014.11.017 25572155

[99] Kulkarni P Dasgupta P Bhat NS Shahryari V Shiina M Hashimoto Y Elevated miR-182-5p Associates with Renal Cancer Cell Mitotic Arrest through Diminished MALAT-1 Expression. Mol Cancer Res (2018) 16(11):1750–60. 10.1158/1541-7786.MCR-17-0762 PMC6214767 30037856

[100] Liu F Chen N Xiao R Wang W Pan Z miR-144-3p serves as a tumor suppressor for renal cell carcinoma and inhibits its invasion and metastasis by targeting MAP3K8. Biochem Biophys Res Communications (2016) 480(1):87–93. 10.1016/j.bbrc.2016.10.004 27717821

[101] Liu F Wu L Wang A Xu Y Luo X Liu X MicroRNA-138 attenuates epithelial-to-mesenchymal transition by targeting SOX4 in clear cell renal cell carcinoma. Am J Trans Res (2017) 9(8):3611–22. PMC5575175 28861152

[102] Okato A Arai T Yamada Y Sugawara S Koshizuka K Fujimura L Dual Strands of Pre-miR-149 Inhibit Cancer Cell Migration and Invasion through Targeting FOXM1 in Renal Cell Carcinoma. Int J Mol Sci (2017) 18(9):1969. 10.3390/ijms18091969 PMC5618618 28902136

[103] Nofech-Mozes R Khella HWZ Scorilas A Youssef L Krylov SN Lianidou E MicroRNA-194 is a Marker for Good Prognosis in Clear Cell Renal Cell Carcinoma. Cancer Med (2016) 5(4):656–64. 10.1002/cam4.631 PMC4831284 26860079

[104] Machackova T Mlcochova H Stanik M Dolezel J Fedorko M Pacik D MiR-429 is linked to metastasis and poor prognosis in renal cell carcinoma by affecting epithelial-mesenchymal transition. Tumor Biol (2016) 37(11):14653–8. 10.1007/s13277-016-5310-9 27619681

[105] Qin Z Wei X Jin N Wang Y Zhao R Hu Y MiR-199a targeting ROCK1 to affect kidney cell proliferation, invasion and apoptosis. Artif Cells Nanomed Biotechnol (2018) 46(8):1920–5. 10.1080/21691401.2017.1396224 29130345

[106] Pan Y-J Wei L-L Wu X-J Huo F-C Mou J Pei D-S MiR-106a-5p inhibits the cell migration and invasion of renal cell carcinoma through targeting PAK5. Cell Death Disease (2017) 8(10):e3155–5. 10.1038/cddis.2017.561 PMC5680926 29072688

[107] Wang D Zhu C Zhang Y Zheng Y Ma F Su L MicroRNA-30e-3p inhibits cell invasion and migration in clear cell renal cell carcinoma by targeting Snail1. Oncol Lett (2017) 13(4):2053–8. 10.3892/ol.2017.5690 PMC5403512 28454361

[108] Wang C Wu C Yang Q Ding M Zhong J Zhang C-Y miR-28-5p acts as a tumor suppressor in renal cell carcinoma for multiple antitumor effects by targeting RAP1B. Oncotarget (2016) 7(45):73888–902. 10.18632/oncotarget.12516 PMC5342021 27729617

[109] Wu A Wu K Li M Bao L Shen X Li S Upregulation of microRNA-492 induced by epigenetic drug treatment inhibits the malignant phenotype of clear cell renal cell carcinoma in vitro. Mol Med Rep (2015) 12(1):1413–20. 10.3892/mmr.2015.3550 25815441

[110] Wang M Gao H Qu H Li J Liu K Han Z MiR-137 suppresses tumor growth and metastasis in clear cell renal cell carcinoma. Pharmacol Rep (2018) 70(5):963–71. 10.1016/j.pharep.2018.04.006 30107346

[111] Xiang C S-p C Ke Y MiR-144 inhibits cell proliferation of renal cell carcinoma by targeting MTOR. J Huazhong Univ Sci Technol [Medical Sciences] (2016) 36(2):186–92. 10.1007/s11596-016-1564-0 27072960

[112] Xu M Gu M Zhang K Zhou J Wang Z Da J miR-203 inhibition of renal cancer cell proliferation, migration and invasion by targeting of FGF2. Diagn Pathol (2015) 10:24–4. 10.1186/s13000-015-0255-7 PMC4419389 25890121

[113] Xu X Liu C Bao J Hypoxia-induced hsa-miR-101 promotes glycolysis by targeting TIGAR mRNA in clear cell renal cell carcinoma. Mol Med Rep (2017) 15(3):1373–8. 10.3892/mmr.2017.6139 28138701

[114] Zhang H Li H miR-137 inhibits renal cell carcinoma growth in vitro and in vivo. Oncol Lett (2016) 12(1):715–20. 10.3892/ol.2016.4616 PMC4907286 27347205

[115] Zhu S Huang Y Su X Mir-451 Correlates with Prognosis of Renal Cell Carcinoma Patients and Inhibits Cellular Proliferation of Renal Cell Carcinoma. Med Sci Monit Int Med J Exp Clin Res (2016) 22:183–90. 10.12659/MSM.896792 PMC4723065 26779781

[116] Zhao X Zhao Z Xu W Hou J Du X Down-regulation of miR-497 is associated with poor prognosis in renal cancer. Int J Clin Exp Pathol (2015) 8(1):758–64. PMC4348935 25755771

[117] Zhang X Xing N-D Lai C-J Liu R Jiao W Wang J MicroRNA-375 Suppresses the Tumor Aggressive Phenotypes of Clear Cell Renal Cell Carcinomas through Regulating YWHAZ. Chin Med J (2018) 131(16):1944–50. 10.4103/0366-6999.238153 PMC6085851 30082525

[118] Sun X Lou L Zhong K Wan L MicroRNA-451 regulates chemoresistance in renal cell carcinoma by targeting ATF-2 gene. Exp Biol Med (Maywood NJ) (2017) 242(12):1299–305. 10.1177/1535370217701625 PMC5476336 28429654

[119] Chan Y Yu Y Wang G Wang C Zhang D Wang X Inhibition of MicroRNA-381 Promotes Tumor Cell Growth and Chemoresistance in Clear-Cell Renal Cell Carcinoma. Med Sci Monit Int Med J Exp Clin Res (2019) 25:5181–90. 10.12659/MSM.915524 PMC6642673 31299041

[120] Long Q-Z Du Y-F Liu X-G Li X He D-L miR-124 represses FZD5 to attenuate P-glycoprotein-mediated chemo-resistance in renal cell carcinoma. Tumor Biol (2015) 36(9):7017–26. 10.1007/s13277-015-3369-3 25861751

[121] Tang K Xu H Prognostic value of meta-signature miRNAs in renal cell carcinoma: an integrated miRNA expression profiling analysis. Sci Rep (2015) 5:10272. 10.1038/srep10272 25974855 PMC4431463

[122] Tusong H Maolakuerban N Guan J Rexiati M Wang WG Azhati B Functional analysis of serum microRNAs miR-21 and miR-106a in renal cell carcinoma. Cancer Biomark (2017) 18(1):79–85. 10.3233/CBM-160676 27814278 PMC13020616

[123] Wang C Ding M Zhu Y-Y Hu J Zhang C Lu X Circulating miR-200a is a novel molecular biomarker for early-stage renal cell carcinoma. ExRNA (2019) 1(1):25. 10.1186/s41544-019-0023-z

[124] Chanudet E Wozniak MB Bouaoun L Byrnes G Mukeriya A Zaridze D Large-scale genome-wide screening of circulating microRNAs in clear cell renal cell carcinoma reveals specific signatures in late-stage disease. Int J cancer (2017) 141(9):1730–40. 10.1002/ijc.30845 28639257

[125] Sage AP Minatel BC Marshall EA Martinez VD Stewart GL Enfield KSS Expanding the miRNA transcriptome of human kidney and renal cell carcinoma. Int J Genomics (2018) 2018. 10.1155/2018/6972397 PMC6051088 30057905

[126] Kurahashi R Kadomatsu T Baba M Hara C Itoh H Miyata K MicroRNA-204-5p: A novel candidate urinary biomarker of Xp11. 2 translocation renal cell carcinoma. Cancer Sci (2019) 110(6):1897. 10.1111/cas.14026 31006167 PMC6549932

[127] Nakamura T Iwamoto T Nakamura HM Shindo Y Saito K Yamada A Regulation of miR-1-mediated connexin 43 expression and cell proliferation in dental epithelial cells. Front Cell Dev Biol (2020) 8:156. 10.3389/fcell.2020.00156 32258035 PMC7089876

[128] Chen B Duan L Yin G Tan J Jiang X miR-381, a novel intrinsic WEE1 inhibitor, sensitizes renal cancer cells to 5-FU by up-regulation of Cdc2 activities in 786-O. J Chemother (2013) 25(4):229–38. 10.1179/1973947813Y.0000000092 23816136

[129] Zhang Q Di W Dong Y Lu G Yu J Li J High serum miR-183 level is associated with poor responsiveness of renal cancer to natural killer cells. Tumour Biol J Int Soc Oncodevelopmental Biol Med (2015) 36(12):9245–9. 10.1007/s13277-015-3604-y 26091793

[130] Xiao W Wang X Wang T Xing J MiR-223-3p promotes cell proliferation and metastasis by downregulating SLC4A4 in clear cell renal cell carcinoma. Aging (2019) 11(2):615–33. 10.18632/aging.101763 PMC6366987 30668544

[131] Baumann V Winkler J miRNA-based therapies: strategies and delivery platforms for oligonucleotide and non-oligonucleotide agents. Future Med Chem (2014) 6(17):1967–84. 10.4155/fmc.14.116 PMC4417715 25495987

[132] Youssef YM White NM Grigull J Krizova A Samy C Mejia-Guerrero S Accurate molecular classification of kidney cancer subtypes using microRNA signature. Eur Urol (2011) 59(5):721–30. 10.1016/j.eururo.2011.01.004 21272993

[133] von Brandenstein M Pandarakalam JJ Kroon L Loeser H Herden J Braun G MicroRNA 15a, inversely correlated to PKCα, is a potential marker to differentiate between benign and malignant renal tumors in biopsy and urine samples. Am J Pathol (2012) 180(5):1787–97. 10.1016/j.ajpath.2012.01.014 22429968

